# Validation of IFMSA social accountability assessment tool: exploratory and confirmatory factor analysis

**DOI:** 10.1186/s12909-023-04121-7

**Published:** 2023-03-01

**Authors:** Özlem Coşkun, Utku Timurçin, Yavuz Selim Kıyak, Işıl İrem Budakoğlu

**Affiliations:** 1 Medical Education and Informatics, Gazi University Faculty of Medicine, Ankara, Turkey; 2 Gazi University Faculty of Medicine, Ankara, Turkey; 3 Faculty of Medicine, Department of Medical Education and Informatics, Gazi University, Gazi Üniversitesi Hastanesi E Blok 9. Kat, Beşevler, Ankara, 06500 Turkey

**Keywords:** Social accountability, Assessment tool, Factor analysis, Medical education

## Abstract

**Background:**

IFMSA Social Accountability Assessment Tool has been developed for medical students by medical students to assess medical schools. However, its psychometric characteristics are unknown since it was developed without any analysis. We aimed to reveal its reliability and validity.

**Methods:**

1122 undergraduate medical students from various years in Gazi University Faculty of Medicine have participated in the study. They have answered the Turkish version of IFMSA Social Accountability Assessment Tool created through a translation process by experts. Exploratory and confirmatory factor analyses were carried out.

**Results:**

Exploratory factor analysis showed that factor loadings were between 0.46 and 0.73 for Factor 1, 0.68 and 0.87 for Factor 2. The two-factor model, which consists of “Community Centeredness” and “Socio-Demographic Characteristics”, was evaluated through confirmatory factor analysis. The goodness-of-fit statistics of the model showed well-fit: CMIN/df 4.46, GFI 0.96, CFI 0.95, RMSEA 0.05, SRMR 0.03. Standardized regression weights were between 0.43 and 0.77.

**Conclusion:**

The tool has acceptable psychometric characteristics, with good reliability and validity. It could be considered as a point of departure for the change in the way of being socially accountable since it enables medical students to explore the weak areas of their medical schools in terms of social accountability.

## Introduction

Social accountability of medical schools has been defined by the World Health Organization (WHO) in 1995 as “the obligation to direct their education, research and service activities towards addressing the priority health concerns of communities, region and/or nation they have a mandate to serve” [1]. When this definition has been adopted by “The Global Consensus for the Social Accountability of Medical Schools” [2], social accountability has attracted more interest year by year for the sake of responding societal needs. Many institutions and networks have strived for improving social accountability of medical schools by organizing global conferences, symposiums, and workshops [3]. Medical school accreditation standards in various countries also included social accountability as a component [4]. A recent global survey of medical school deans and program directors showed that many medical schools have made social accountability a key part of their policies and mission statements [5]. Furthermore, certain medical schools have received recognition for their adherence to principles of social accountability through the conferral of excellence awards by the Association for Medical Education in Europe (AMEE) [6].

Since it is difficult to manage without measuring, medical schools need tools to measure their social accountability [7] in this era of growing interest. Measurement of progress is one of the nine key drivers of social accountability [8]. However, it is a fact that the task of measuring this kind of concept is complex [9]. A study on the theoretical perspective of social accountability showed that there is no single universal understanding of social accountability [10]. It depends on cultural and contextual factors, as it has been perceived differently in, for example, Japan [11] and Egypt [12].

Expert panels [13, 14] and evaluation frameworks [15–17] have been utilized in order to evaluate and promote social accountability. Furthermore, some specific tools such as an inventory for evaluating social accountability principles in problem-based learning scenarios have been developed [18, 19]. Even if these tools and approaches are useful for evaluating social accountability considering different specific purposes, none of them showed psychometric evidence for validity. Only “The Social Accountability Instrument for Latin America” reported psychometric characteristics of the tool by conducting factor analysis but it is specific to the Latin American context [20]. The literature stresses that more studies are needed for further development and validation of instruments to assess social accountability within medical education institutions [21, 22].

Among all the tools and frameworks, the IFMSA (International Federation of Medical Students Associations) Student Toolkit deserves particular attention because it has been developed by students for students [23]. As a scoping review showed that assessments for social accountability were mostly based on experts’ perspectives [24], student voice in the evaluation of social accountability still needs to be heard. IFMSA Student Toolkit would be a useful tool for filling this gap. By using this tool, given that medical student participation is crucial for developing socially accountable medical schools [25], they could play an important role for paving the way for change by raising awareness in terms of social accountability. However, the development process of the tool did not include any validity and reliability analysis (Jeremy Glasner, January 6, 2020, personal communication via e-mail). It is clear that there is a need to explore its psychometric characteristics in order to use it confidently.

The objectives of this study are as follows:

1. To reveal the psychometric characteristics of the Turkish version of the IFMSA Social Accountability Assessment Tool by carrying out exploratory and confirmatory factor analysis.
2. To determine how medical students at Gazi University assess the social accountability of their medical school.

## Methods

### Participants

This study was conducted in Gazi University Faculty of Medicine, Ankara, Turkey. In total, there were 2649 undergraduate medical students of the six-year-long medical degree curriculum in the period of 2020–2021. Based on the convenience sampling method, out of 2649 medical students, 1122 (42.3%) of them have filled the survey form entirely. Of them, 215 (19.2%) were Year-1, 131 (11.7%) were Year-2, 210 (18.7%) were Year-3, 156 (13.9%) were Year-4, 130 (11.6%) were Year-5, and 280 (25.0%) were Year-6 students. Year-1 to Year-4 students had limited experience in the clinical environment due to remote education led by COVID-19 pandemic, Year-5 and Year-6 students had completed several clerkships before the onset of the pandemic.

### Instrument

IFMSA Social Accountability Assessment Tool has been developed by international medical students who created a common understanding of social accountability in their Standing Committee [23]. They shared their work to inspire and aggravate the importance of social accountability among medical students, while also providing a student-centered perspective. In other words, it was aimed to encourage medical students to assess their medical schools in terms of social accountability, and to identify the potential areas for improvement by reflecting on the results.

The tool consists of 12 questions each with four options that were provided to rate the performance of the school from zero to three points. Zero implicates “None”, one “Somewhat”, two “Good”, and three “Excellent”.

Each question had its own “further explanation” in the annex part. The annex part delves deeper into the topic, providing the primary rationale behind the initial inquiry and supplying additional questions for students to discern areas of deficiency and gather evidence to support their advocacy efforts.

Totally 36 points in maximum come from 12 questions [23]:

- 0–8 points: “There is a need to start a conversation with classmates and school to start building social accountability at the school.”
- 9–17 points: “The school has some social accountability strategies but there is still need to advocate these existing strategies.”
- 18–26 points: “The school is doing well, now it is time to look for areas of weakness and ways to advocate for the improvement of social accountability.”
- 27–36 points: “The school has a strong social accountability foundation. Keep advocating for continued growth and leadership in the field.”

#### Translation process and pilot

The research team translated the questions into Turkish. Due to practical reasons, we did not add the annex part, which consists of two-page-long information, to the survey form. But we added explanations derived from the annex part. These explanations included one to four sentences for each question, and they were placed below each question. In order to prevent misinterpretation or mistranslation as much as possible, expert opinion has been gathered from four professors in medical education field in Turkey via e-mail. The questions and explanations were then retranslated into English to spot if there are any possible misperceptions that might arise. It denoted the conclusion of the initial phase of the drafting process.

After consulting with experts to determine the most accurate forms of translation, we created two pilot groups. In the first group, we had five voluntary students who acknowledge the concept of social accountability (who are working in national and/or international student associations, faculty committees, or as IFMSA certified trainers). They were chosen to give feedback prior to finalizing our survey form. In the second group, we had five students who had not heard anything related to the concept of social accountability. We aimed to see how these two groups understand the questions and the meaning behind every question, and especially to get a peer review with student opinion. Thus, we aimed to prevent any misconceptions during the survey. We have updated the survey considering their feedback. The form we used (Turkish) can be accessible by contacting the corresponding author.

#### Analysis

Exploratory Factor Analysis: We conducted exploratory factor analysis using SPSS v.22.0 for Windows. The data acquired from 1122 undergraduate medical students was analyzed. To determine whether the data is adequate to conduct factor analysis, we have carried out two examinations. The first one we considered was the evaluation of the correlation matrix. Conducting factor analysis does not make sense if there is no correlation between items over 0.30 [26]. Correlation values (Spearman’s Rho) between the items were higher than 0.30, which means that there is no inadequacy. Subsequently, Bartlett’s Test of Sphericity and the Kaiser-Meier-Olkin Measure of Sampling Adequacy (KMO MSA) results were evaluated. Bartlett’s Test of Sphericity was significant (< 0.05) and KMO MSA was 0.92. Both of the results showed that the data has no inadequacy to carry out factor analysis [26].

To identify the number of factors, we employed three strategies: (a) Eigenvalue cut-off rule, (b) the “elbow” joint in the scree plot, and (c) meaningfulness of factors [26, 27]. We used Principal Component Analysis and Direct Oblimin as the extraction and rotation methods, respectively. Direct Oblimin, which is an oblique rotation technique, was the best technique for our study since factor intercorrelation is a norm for the studies in social sciences [28]. We accepted 0.40 level as a factor loading threshold to consider that a factor is stable [29].

Confirmatory Factor Analysis: We used AMOS 24 software to conduct confirmatory factor analysis. The method we used was the maximum likelihood method. The data has not violated the assumptions of this method, which are a large sample size and multivariate normal distribution of variables [30]. The model fit was evaluated using these statistics and indices: (a) minimum discrepancy per degree of freedom (CMIN/df) should be lower than 5 [31], (b) the Goodness of Fit Index (GFI) and (c) the Comparative Fit Index (CFI) should be greater than 0.90 [32, 33], (d) the Root Mean Square Error of Approximation (RMSEA) should be less than 0.05 [33], (e) the Standardized Root Mean Square Residual (SRMR) should be less than 0.08 to indicate good fit [34]. Factor loading values above or close to 0.70 are better to explain the structure [35].

Social Accountability of the School: We used SPSS v.22.0 for Windows to analyze the opinions of the participants about the social accountability of the school. We calculated the mean value and standard deviation for each item, and the overall score. We also compared the scores acquired from Year-5-6 and Year-1 to Year-4 students using Independent Samples T-Test.

### Ethical considerations

The initial step of the research process began with obtaining permission to work with IFMSA Social Accountability Assessment Tool from the respective Liaison Officer. Ethical approval for this research was granted on March 3, 2020 by the Gazi University Ethical Committee (Code: 2020 − 175) prior to participation in this study. All individuals provided written consent.

## Results

Cronbach’s alpha level of the tool that consists of 12 questions was 0.87 (95% confidence intervals: 0.85–0.88).

### Exploratory factor analysis

The structure was accepted as two-factor since (a) the eigenvalues of Factor 1 and Factor 2 were 5.05 and 0.99, respectively, (b) the “elbow” joint of the scree plot was on Factor 2, and (c) the factors were meaningful. Furthermore, the two factors explained 50.27% of variance cumulatively. While factor loadings were between 0.46 and 0.73 for Factor 1, the loadings were 0.68 and 0.87 for Factor 2 (Table 1). Thus, Factor 1 was named “Community Centeredness”, and Factor 2 was named “Socio-Demographic Characteristics”.

**Table 1:** Factor loadings from exploratory factor analysis using direct oblimin rotation

	Item	Factor 1	Factor 2
1	Does your institution have a clear social mission (statement) around the communities that they serve?	**0.73**	-0.06
2	Does your curriculum reflect the needs of the population you serve?	**0.65**	0.12
3	Does your school have community partners and stakeholders who shape your school?	**0.64**	0.01
4	Do you learn about other cultures and other social circumstances in medical context in your curriculum?	**0.61**	0.14
5	Do the places/locations you learn at in practice include the presence of the populations that you will serve?	**0.46**	0.29
6	Are you required to do community based learning (opposed to only elective opportunities)?	**0.59**	0.22
7	Does your class reflect the socio-demographic characteristics of your reference population?	-0.02	**0.87**
8	Do your teachers reflect the socio-demographic characteristics of your reference population?	0.20	**0.68**
9	Does your learning experience also provide an active service to your community?	**0.57**	0.24
10	Does your school have community based research?	**0.68**	0.02
11	Does your school encourage you to undertake generalist specialties (e.g. family medicine, general practice)?	**0.64**	-0.22
12	Does your school have a positive impact on the community?	**0.72**	-0.03

### Confirmatory factor analysis

**Fig. 1:**
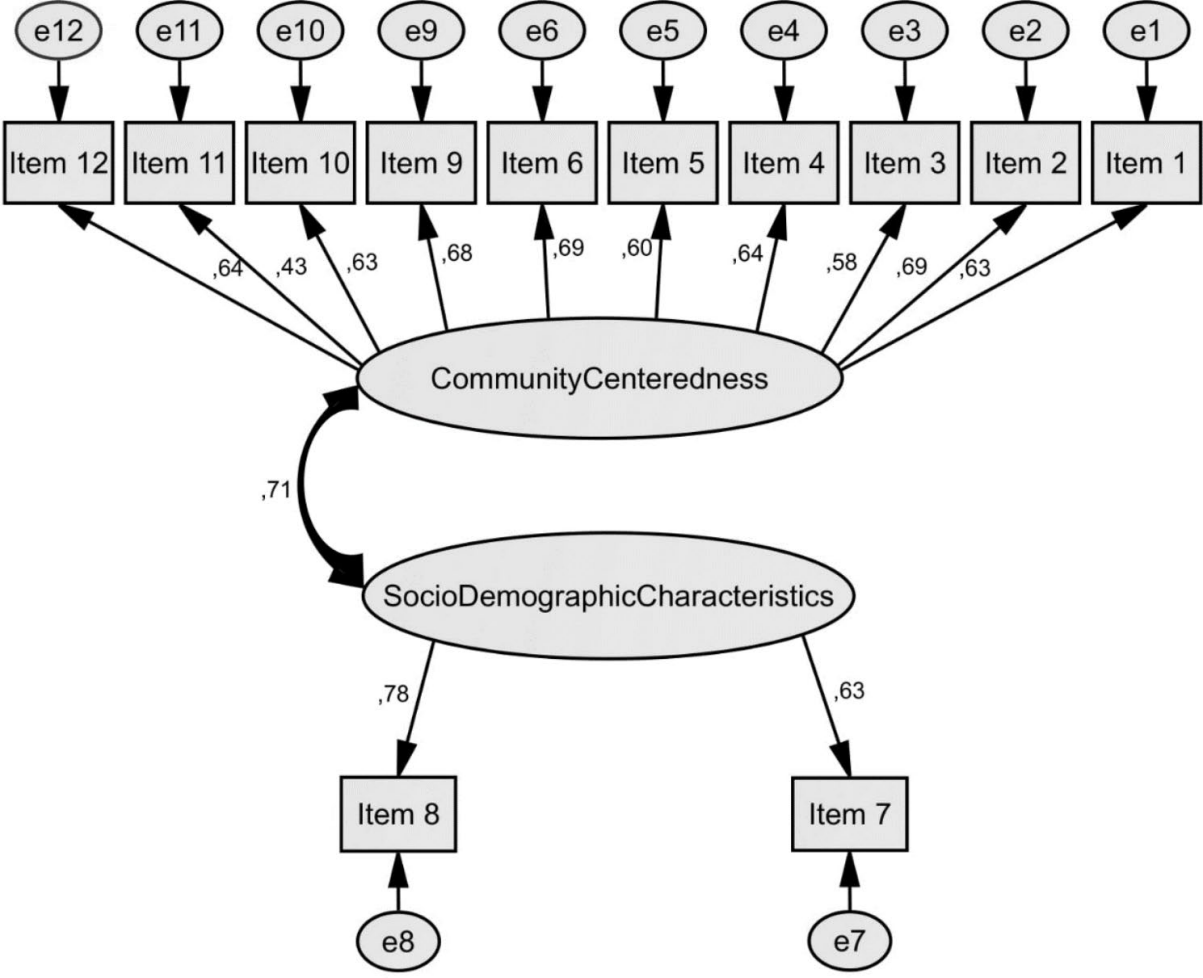
Two-factor model diagram, and standardized regression weights of the items

The two-factor model was established considering the results of the exploratory factor analysis (Fig. 1). The goodness-of-fit statistics of the model were as following: CMIN/df was 4.46, GFI was 0.96, CFI was 0.95, RMSEA was 0.05, SRMR was 0.03. These statistics indicated that it fitted well.

In Fig. 1, the twelve observed variables are represented by the squares. Two ovals at the center represent the two factors. Error terms represented by the ovals contained little “e” on them. The arrows symbolize the effects of the elements. The two-way arrow and the value on the arrow reveal factor correlation. The values are trait correlations corrected for unreliability. Standardized regression weights were between 0.43 and 0.77 (Table 2). All standardized regression weights were close to 0.70 but two items (Item 3 and Item 11) were not. It indicated that the two-factor model explained the majority of the items.

**Table 2:** Standardized regression weights, confirmatory factor analysis

Items		Weights
1	Does your institution have a clear social mission (statement) around the communities that they serve?	0.63
2	Does your curriculum reflect the needs of the population you serve?	0.69
3	Does your school have community partners and stakeholders who shape your school?	0.58
4	Do you learn about other cultures and other social circumstances in medical context in your curriculum?	0.64
5	Do the places/locations you learn at in practice include the presence of the populations that you will serve?	0.60
6	Are you required to do community based learning (opposed to only elective opportunities)?	0.69
7	Does your class reflect the socio-demographic characteristics of your reference population?	0.63
8	Do your teachers reflect the socio-demographic characteristics of your reference population?	0.78
9	Does your learning experience also provide an active service to your community?	0.68
10	Does your school have community based research?	0.63
11	Does your school encourage you to undertake generalist specialties (e.g. family medicine, general practice)?	0.43
12	Does your school have a positive impact on the community?	0.64

### Social accountability level of the School

The overall score calculated using 12 questions was 18.82 ± 5.88. Table 3 presents item-based values. The lowest score was on Item 11 that is about encouraging the medical students to work as a generalist. The highest one was Item 12 that implies the positive impact of the school on the community.

**Table 3:** Item-based mean values (M) and standard deviations (SD).

No	Item	M	SD
1	Does your institution have a clear social mission (statement) around the communities that they serve?	1.56	0.71
2	Does your curriculum reflect the needs of the population you serve?	1.82	0.67
3	Does your school have community partners and stakeholders who shape your school?	1.50	0.72
4	Do you learn about other cultures and other social circumstances in medical context in your curriculum?	1.31	0.78
5	Do the places/locations you learn at in practice include the presence of the populations that you will serve?	1.83	0.80
6	Are you required to do community based learning (opposed to only elective opportunities)?	1.62	0.75
7	Does your class reflect the socio-demographic characteristics of your reference population?	1.74	0.79
8	Do your teachers reflect the socio-demographic characteristics of your reference population?	1.45	0.79
9	Does your learning experience also provide an active service to your community?	1.61	0.77
10	Does your school have community based research?	1.60	0.74
11	Does your school encourage you to undertake generalist specialties (e.g. family medicine, general practice)?	0.77	0.86
12	Does your school have a positive impact on the community?	1.96	0.73

The overall score of Year-5 and Year-6 students (n = 712, M = 18.30, SD = 5.77) was significantly lower than the overall score of Year-1 to Year-4 students (n = 410, M = 19.11, SD = 5.93), t(1120) = 2.22 p = 0.026.

## Discussion

In this study, we aimed to reveal psychometric characteristics of IFMSA Social Accountability Assessment Tool since it was developed without conducting any psychometric study. The exploratory factor analysis showed that the structure includes two factors that are “Community Centeredness” and “Socio-Demographic Characteristics”. The two-factor model established according to exploratory factor analysis was analyzed through carrying out confirmatory factor analysis. The model fitted well and explained the majority of the items. Only one item, which is Item 11, had a relatively low regression weight but we did not exclude it since encouraging medical students to work as a generalist is crucial in terms of social accountability [16]. Altogether, the analyses showed that the Turkish version of the tool has reasonable psychometric characteristics, with good internal reliability and structural validity. This is among the first studies in the literature that provides psychometric validity evidence for assessment of social accountability, just as the tool developed for the Latin American context provided the results of a factor analysis [20].

The tool, now, could be used with more confidence by medical students to assess the social accountability of their medical schools. Students are considered among the actors that can drive organizational change in terms of social accountability [36]. Through the tool, they could spot the weak areas and subsequently take action for the remedies. Since the tool provides ways on how to approach quality improvement processes, students who are interested could be recommended to read the entire document. By doing so, medical students have an opportunity to explore a few practical methods regarding how to take action, such as “building capacity, raising the issue, a letter to your student organization or school administration, and social media”. It may lead social accountability assessment to become more inclusive in terms of medical student participation. It may also contribute to solving the problem of social accountability assessments being heavily reliant on expert perspectives alone [24].

In our context, the results showed that the school got 18.82 out of total of 36 points. The score is not perfect but good. The advice of the tool for our students is “Your school is doing well, look for areas of weakness and ways to advocate to improve social accountability”. It is not surprising for one of the prominent Turkish medical schools to have a good level of social accountability since the Turkish medical schools put more emphasis for a while on improving social accountability under the guidance of The Association of Evaluation and Accreditation of Medical Education Programs (TEPDAD), which is “the second agency to be awarded the Recognition Status by the World Federation for Medical Education (WFME)” [37]. A solid sign of it is that TEPDAD, by including a diverse and wide range of participants, developed a social accountability framework that meets the local needs for Turkish medical schools [38]. However, the medical schools in Turkey still have a lot of room to grow by means of social accountability. Considering the key actors of the Turkish medical education community have a strong motivation to improve social accountability of the schools [39], the medical students have the potential to trigger a huge progress by taking action, guided by the assessment of their medical schools through this tool.

The most useful aspect of the tool is that it exposes the weakest side of the school in terms of social accountability. It could be considered useful since it allows medical students to focus on an area for improvement to initiate change. For instance, the weakest area of our school was the lack of encouragement to work in rural areas or primary healthcare facilities, just as a study conducted using IFMSA tool showed that the weakest area in a medical school in Saudi Arabia is the same [40]. The result is consistent with the recent analysis of the specialization trends of Turkish medical students from 1987 to 2017 that shows the students focused on to work as a specialist who has high salary with low malpractice risk in secondary or tertiary healthcare facilities [41] instead of working as a primary care physician. To break this trend, a movement created by the students would be effective. Now it is time for our medical school to provide opportunities to students to collaborate in order to lead the change.

Our study has several limitations. The first limitation is that it includes the data from only one medical school from Turkey, therefore it may not be generalizable. Furthermore, even if it showed a valid structure for a Turkish context, other languages and contexts still need to be studied since it is a matter of cultural, social, political, and organizational background [10]. Another limitation is that the tool covers only a portion of the structure of social accountability. Its extent could have been enhanced by adding more items but we did not want to make any changes in order not to affect the authenticity of the tool. The last limitation we should mention is that even if the tool provides the cut-off values for interpretation of the overall score, we have not conducted any analysis on the validity of these threshold values. Future studies focusing on determining these values are needed. Apart from that, future studies are expected to be carried out to show how the use of the tool impacts the practice in the long term since evidence of impact from the concept of ‘‘social accountability’’ as an entity, or even enhanced awareness and change in attitudes including the potential impact on future practice is limited [42].

## Conclusion

IFMSA Social Accountability Assessment Tool has been developed by medical students for medical students to assess their medical schools but there was not any psychometric study conducted about this tool before. The current study has revealed the psychometric characteristics of its Turkish version by conducting exploratory and confirmatory factor analysis. The results showed that the tool has acceptable psychometric characteristics, with good reliability and validity. In the context of our medical school, the tool recommended our students to discern areas of deficiency and to explore ways to advocate for improvements in social accountability, with the understanding that their school is overall performing well. Since the tool allows medical students to explore the weak areas in terms of social accountability, it could provide a starting point for the change in order to create more socially accountable medical schools. We hope our study could bring about paving the way for change.

## Data Availability

The datasets used and/or analysed during the current study available from the corresponding author on reasonable request. However, the Turkish version of the assessment tool is available in a public repository at 10.5281/zenodo.7528107.
